# SiNPs induce ferroptosis in HUVECs through p38 inhibiting NrF2 pathway

**DOI:** 10.3389/fpubh.2023.1024130

**Published:** 2023-02-08

**Authors:** Xiaojun Jiang, Huiqian Gao, Yunchang Cao, Shuting Chen, Fangfang Huang, Yashi Feng, Yuqi Zhang, Shaolong Feng

**Affiliations:** ^1^The Guangxi Key Laboratory of Environmental Exposomics and Entire Lifecycle Health, School of Public Health, Guilin Medical University, Guilin, China; ^2^The School of Public Health, University of South China, Hengyang, China; ^3^The Department of Molecular Biology, School of Intelligent Medicine and Biotechnology, Guilin Medical University, Guilin, China; ^4^The State Key Laboratory of Organic Geochemistry, Guangzhou Institute of Geochemistry, Chinese Academy of Sciences, Guangzhou, China

**Keywords:** ferroptosis, sillica nanoparticle (SiNP), endothelial cell (EC), p38, Nrf2

## Abstract

**Introduction:**

Despite of growing evidence linking silica nanoparticles (SiNPs), one of the global-top-three-produced and -used nanoparticle (NP), to human health risks, there remain many knowledge gaps over the adverse effects of SiNPs exposure on cardiovascular system and the underlying molecular mechanisms.

**Methods:**

In this study, the ferroptotic effects of SiNPs (20 nm; 0, 25, 50, and 100 μg/mL) on human umbilical vein endothelial cells (HUVECs) and the possible molecular mechanism were studied with the corresponding biochemical and molecular biology assays.

**Results and discussion:**

The results showed that at the tested concentrations, SiNPs could decrease HUVEC viability, but the deferoxamine mesylate (an iron ion chelator) might rescue this reduction of cell viability. Also, increased levels of intracellular reactive oxygen species and enhanced mRNA expression of lipid oxidation enzymes (ACSL4 and LPCAT3) with increase in lipid peroxidation (malondialdehyde), but decreased ratios of intracellular GSH/total-GSH and mitochondrial membrane potential as well as reduced enzymatic activities of anti-oxidative enzymes (CAT, SOD, and GSH-PX), were found in the SiNPs-treated HUVECs. Meanwhile, increase in p38 protein phosphorylation and decrease in NrF2 protein phosphorylation with reduced mRNA expressions of downstream anti-oxidative enzyme genes (CAT, SOD1, GSH-PX, and GPX4) was identified in the SiNPs-exposed HUVECs. These data indicated that SiNPs exposure might induce ferroptosis in HUVECs *via* p38 inhibiting NrF2 pathway. Ferroptosis of HUVECs will become a useful biomarker for assessing the cardiovascular health risks of environmental contaminants.

## 1. Introduction

The increasingly extensive production and widespread usage of nanomaterials have resulted in myriad engineered nanoparticles (ENPs) entering into the eco-environments and human occupational and living environments (1–5). The exposure of humans and eco-environments to these ENPs has been increasing through all possible routes over their life cycle (1–5). Thus, globally ever-increasing concerns have been raised on the potential adverse effects of ENPs exposure on humans and eco-environments. But, currently, their impacts on human health and eco-environmental safety, as well as the underpinning biological mechanisms, are still far from being well understood (6).

Silica nanoparticles (SiNPs), one of the most extensively-produced ENPs, have been widely applied to various areas, including but not limited to, foods, building materials, biomedical and biotechnological field, and electronic and chemical industries (1, 7). SiNPs have also been ranked in the top three global nanomaterial-based consumer products (1, 7). With the increasing production and application, the exposure of SiNPs to people has been increasing through all possible routes. Notably, following the exposure of inhalation, ingestion or skin, SiNPs can easily pass through the various physiological barriers of mammas and enter the systemic circulation and in turn affect a variety of target organs (8, 9). In addition, SiNPs may be injected directly into the bloodstream in the usages of medicine (10). Consequently, the cardiovascular system is directly exposed to these NPs before they reach other target organs. Thus, their potential adverse effects on the cardiovascular system deserve great concerns. Currently, however, research focusing on their potentials to damage cardiovascular health is relatively limited, in particular, the underpinning biolgical mechanisms have not yet been fully clarified (2, 11).

Atherosclerosis (AS) is an important common pathophysiological basis of cardiovascular diseases (CVDs) (12, 13). Modern medical insights into the fundamental mechanism of AS have shown that dysfunction and impairments of endothelial cells (ECs) lining the innermost layer of the cardiovascular system are not only a critical initial step of AS, but also involved in both the progression of plaques and occurrence of atherosclerotic complications, ultimately resulting in CVDs (12, 14). Ferroptosis is an iron-dependent form of programmed cell death driven by abnormal lipid peroxidation and redox imbalance, which are linked to the pathogenesis of AS (15). Therein, ferroptosis of ECs was indicated to be an important pathological process involved in AS and its complications (16). Our prior works have shown that SiNPs are able to pose multiple toxicities to human umbilical vein endothelial cells (HUVECs), a model cell which is widely used to study the possible molecular mechanisms underlying the pathophysiological processes of AS (1, 17). Up to now, however, it's yet unclear both whether SiNPs exposure may induce ferroptosis in ECs and what the underlying molecular mechanisms are.

In this study, therefore, we firstly determined the potential of SiNPs to induce ferroptosis in HUVECs in culture. After that, the underlying molecular mechanism was probed through measuring the mRNA or protein expression of critical genes involved in the ferroptotic pathway. The aim is to gain an extended understanding in the ferroptotic effects of SiNPs exposure on HUVECs as well as the possible molecular mechanism, in order to offer scientific data to assess their potential risks to public cardiovascular health.

## 2. Materials and methods

### 2.1. SiNPs, chemicals, agents and antibodies

Amorphous SiNPs (20 ± 5.6 nm, their characteristic features were documented in S1) were bought from Jiangsu XFNANO Materials Tech Co., Ltd (Nanjing, China). The chemicals, agents and test kits, primers for RT-qPCR, and antibodies were listed in Supplementary Tables S1–S3, respectively.

### 2.2. Cell culture and SiNPs exposure

HUVECs and their culture conditions have been detailly described in our prior papers (1, 18). The exponentially growing cells were exposed to suspensions of SiNPs (final concentrations: 0, 25, 50, and 100 μg/mL, freshly made by ultrasonic dispersion) in full medium of cell culture to perform the following assays. Triplicate in each dose were prepared.

### 2.3. Cell viability assay

After being seeded in 96-well microplates at a density of 5,000 cells/well for 24 h, HUVECs were treated with SiNPs suspensions (0, 25, 50, and 100 μg/mL) for 24, 48, and 72 h, respectively. The viabilities of HUVECs were determined with the Cell Counting Kit-8 (CCK-8) according to the standardized protocol (17, 19). 10 μl CCK-8 reagent was added into each well and incubated at 37°C in the CO_2_ incubator for 4 h. The light absorbance was determined at 450 nm with a Thermomax microplate reader (Menlo Park, USA).

Then, the viabilities of HUVECs respectively exposed to four groups of SiNPs and/or deferoxamine mesylate (DFO) (SiNPs 25 μg/mL, DFO 28 μg/mL, SiNPs 25 μg/mL + DFO 28 μg/mL) and negative control for 24 h were determined.

### 2.4. RT-qPCR

After treating HUVECs with four groups (negative control, SiNPs 25 μg/mL, DFO 28 μg/mL, SiNPs 25 μg/mL + DFO 28 μg/mL) for 24 h, the total RNAs of HUVECs were extracted with the FastPureCell/Tissue Total RNA Isolation Kit and subsequently were reversely transcribed to cDNAs with the HiScript III 1st Strand cDNA Synthesis Kit according to their manufacturer's protocols. RT-qPCR was performed to quantitatively measure the copy numbers of cDNAs of target genes (their primers were listed in Supplementary Table S2) with the 2 × ChamQ Universal SYBR qPCR Master Mix in the 7,500 Fast Real-Time PCR system (20). The relative levels of mRNA expression of target genes were normalized to that of β-actin using the 2^−ΔΔCt^ method (20).

### 2.5. Intracellular ROS assay

After treatment with suspensions of SiNPs (0, 25, 50, and 100 μg/mL) for 24 h, the levels of intracellular reactive oxygen species (ROS) in HUVECs were detected with a ROS assay kit, which was based on the fluorescent probe 2,7-dichlorofuorescin diacetate (DCFH-DA), according to the standardized protocol (1, 18). The morphology of cells was photographed with an inverted fluorescene microscope qualitatively (Nikon, Japan) and the emission of fluorescence (at 530 nm) was quantified by a Thermo Fisher Flurescence spectrophotometer (Thermo Fisher, USA) (21). The levels of intracellular ROS were expressed directly in the manner of values of mean fluorescence intensity (MFI) measured.

### 2.6. GSH and total GSH assay

Following the treatment of SiNP suspensions (0, 25, 50, and 100 μg/mL) for 24 h, the levels of intracellular GSH and total GSH (T-GSH) in HUVECs were detected respectively with a GSH assay kit and a total GSH assay kit according to their standardized protocols, which use catalytic GSH to reduce 5,5'-dithiobis(2-nitrobenzoic acid) (DTNB) to TNB (1, 18). Briefly, after the protein removal, the extracts of cells were used to determine the levels of GSH and T-GSH respectively with a Thermomax microplate reader (Menlo Park, USA) to measure the absorbance at 412 nm under the procedure of these kits.

### 2.7. MDA assay

The levels of malondialdehyde (MDA), an important biomarker of lipid peroxidation, were measured with a MDA Detection Kit according to the manufacturer's protocol, which bases on the reaction between MDA and thiobarbituric acid to form a red compound that has a largest absorbance at 535 nm (21).

### 2.8. MMP assay

The changes of mitochondrial membrane potential (MMP) were monitored through the fluorescence probe JC-1 within the MMP assay kit according to the manufacturer's protocol (22). Briefly, following 500 μl working solution being added to the cells, the cells were incubated at 37°C in the CO_2_ incubator for 15 min. After washing with incubation buffer, fluorescence intensities of cells were observed with a fluorescence microscope (Nikon, Japan).

### 2.9. Activity assay of anti-oxidative enzymes

Catalase (CAT) activity was determined by a CAT Detection kit according to the manufacturer's protocol, which analyze the rate of CAT-mediated H_2_O_2_ decomposition at 240 nm (23). The activities of CAT were presented as U/mg protein.

Activity of superoxide dismutase (SOD) in HUVECs was detected through a SOD Detection kit according to the manufacturer's protocol (24). The principle is that SOD cleans the superoxide anion produced by xanthine oxidase oxidizing xanthine to block up superoxide anion reducing azoblue tetrazole to form blue methyl, which has the largest light absorption at 560 nm. The activities of SOD were expressed as U/mg protein.

Glutathione peroxidase (GSH-PX) activity was measured with a GSH-PX Detection kit according to the manufacturer's instructions (24). The GSH-PX is able to catalyze the oxidation reaction between GSH and benzoic acid chromogenic to produce yellow anion which has the largest light absorption at 422 nm. The activities of GSH-PX were documented as U/mg protein.

### 2.10. Western blot

The relative levels of target proteins were determined with the western blot according to the standardized procedure, which were detailly described in our prior papers (17–19, 25, 26). Briefly, after treating HUVECs with suspensions of SiNPs (0, 25, 50, and 100 μg/mL) for 24 h, the protein extracts were prepared. Following separation on SDS-PAGE gels, the proteins were transferred to the PVDF films for western blot. After the incubation of primary antibodies for target proteins (Supplementary Table S3) and subsequently incubation of HRP-conjugated second antibodies, the films were developed with a chemiluminescence ECL kit and measured with Tanon 6,100 Chemiluminescent Imaging System. Relative levels of the target proteins to the β-actin were expressed.

### 2.11. Statistical analysis

The data was present in the manner of Mean ± SE (*n* = 3). One-way analysis of variance (Bonferroni *post hoc* test) for multiple group comparison were used and 2 × 2 factorial analysis can be used for analyzing the main effect, interaction and individual effect of SiNPs and DFO on viability, ACSL4, LPCAT etc. Statistical significance was accepted at *p* < 0.05. SPSS Statistics 19.0 was the statistical software.

## 3. Results

### 3.1. Cytotoxicity

**Figure 1:**
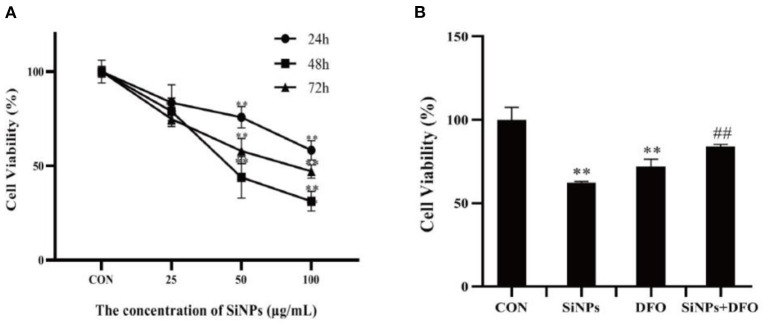
The viabilities of HUVECs under SiNPs exposure. **(A)** The time- and dose-dependent viabilities of HUVECs exposed to SiNPs. **(B)** The DFO rescue the viabilities of HUVECs exposed to SINPs. [The negative control: con; *p < 0.05; **p < 0.01, compared with the con; ^##^*p* < 0.01, compared with the SiNPs, *n* = 3].

As illustrated in Figure 1A, with the increased concentrations and durations of SiNPs exposure, the viabilities of HUVECs were found to significantly decrease, presenting a clearly dose-dependent effect relationship. Interestingly, the DFO, an iron ion chelator that can inhibit the uptake of iron ion by cells, was able to clearly increase the viability of HUVECs when they were simultaneously treated with the DFO and SiNPs (Figure 1B), suggesting that the iron ion has taken an important role in this SiNPs-mediated cytotoxicity to HUVECs, which is a key feature of cell ferroptosis.

### 3.2. Expression of key genes involved in ferroptosis pathway

**Figure 2:**
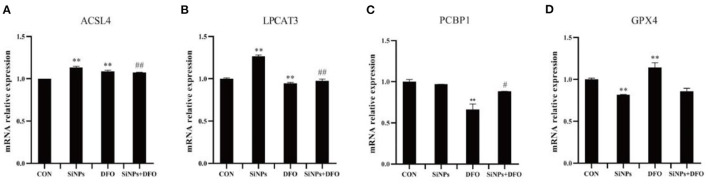
The mRNA expression of key genes involved in ferroptotic pthway. **(A)** The relative mRNA expression of ACSL4. **(B)** The relative mRNA expression of LPCAT3. **(C)** The relative mRNA expression of PCBP1. **(D)** The relative mRNA expression of GPX4. [**p < 0.01, compared with the con; ^#^*p* < 0.05, ^##^*p* < 0.01, compared with the SiNPs, *n* = 3].

To further confirm that the occurrence of ferroptosis in this SiNPs-mediated cytotoxicity to HUVECs, the mRNA expressions of several key genes involved in the modulation of ferroptosis pathway were determined by RT-qPCR. Both ACSL4 (acyl-CoA synthetase long-chain family member 4) and LPCAT3 (lysophosphatidylcholine acyltransferase 3), are two critical enzymes involved in phospholipid metabolism (particularly in the oxidation of polyunsaturated fatty acids) and contribute to ferroptosis (16, 27). The results showed that SiNPs exposure could significantly enhanced the mRNA expressions of both ACSL4 and LPCAT3 in HUVECs, but the DFO was able to decrease their mRNA expressions in HUVECs when they were simultaneously treated with DFO + SiNPs (Figures 2A, B).

PCBP1 (poly (PC) binding protein 1) can bind to Fe^2+^ to generate ferritin, which regulates the availability of intracellular Fe^2+^ (16). The SiNPs exposure did not alter the mRNA expression of PCBP1 in HUVECs, but DFO was able to significantly reduce its mRNA expression in HUVECs (Figure 2C).

GPX4 (glutathione peroxidase 4), an enzyme which transforms lipid hydroperoxides into nontoxic lipid alcohols, is able to effectively abate the impairment mediated by oxidized lipids to biomembrances of cells (16, 27). The results showed that SiNPs exposure could significantly decrease the mRNA expression of GPX4, and that the DFO was able to increase its mRNA expression in HUVECs (Figure 2D).

Collectively, these results suggested that SiNPs exposure might not only increase the mRNA expressions of the key genes (such as ACSL4 and LPCAT3) that can contribute ferroptosis, but also decrease the mRNA expression of anti-ferroptosis gene, GPX4, to induce ferroptosis in HUVECs. Therein, the bioavailability of intracellular Fe^2+^ takes a critical role in this pathophysiological process.

### 3.3. Intracellular oxidative conditions

**Figure 3:**
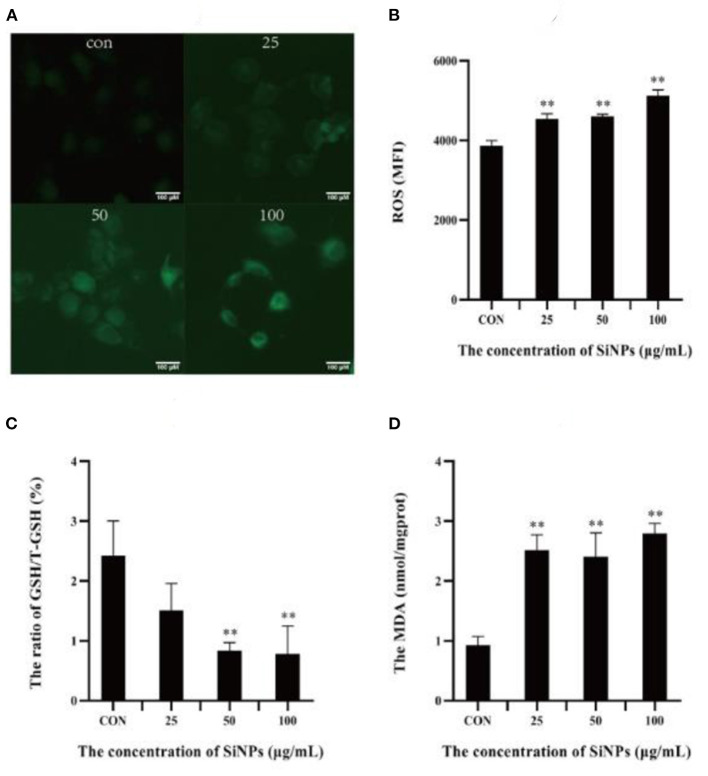
The levels of ROS, MDA and ratios of GSH/T-GSH in HUVECs. **(A)** The intensity of intracellular green fluorescence. **(B)** The relative levels of intracellular ROS. **(C)** The ratio of GSH/total GSH. **(D)** The levels of MDA. [**p < 0.01, compared with the con, *n* = 3].

A specific hallmark of ferroptosis is intracellular redox imbalance and lipid peroxidation with accumulation of lipid hydroperoxides (such as MDA) (16). So the intracellular ROS level, ratio of GSH/total GSH (T-GSH) and content of MDA were detected in HUVECs under SiNPs exposure. The results showed that clearly enhancing levels of intracellular ROS were produced in HUVECs with the elevating concentrations of SiNPs exposure (Figures 3A, B), showing a positive dose-effect relationship (*P* < 0.01). Correspondingly, the ratios of GSH/T-GSH were decreased with the increased concentrations of SiNPs treated (Figure 3C). These results suggested SiNPs exposure induced redox imbalance in HUVECs. At the same time, the levels of MDA were also increasing with the elevating concentrations of SiNPs exposure (Figure 3D), presenting a similar manner of dose-effect relationship. These data further confirmed that SiNPs exposure could induce ferroptosis in HUVECs.

**Figure 4:**
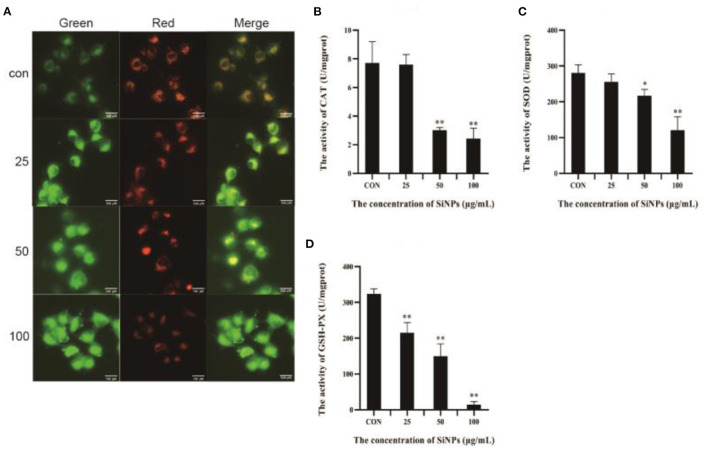
The impairment of mitochndria and activities of anti-oxidative enzymes. **(A)** The changes of mitochondrial membrane potential (intensity of the red fluorescence is positive correlation to the height of mitochondrial membrane potential). **(B)** The activities of CAT. **(C)** The activities of SOD. **(D)** The activities of GSH-PX. [*p < 0.05, **p < 0.01, compared with the con, *n* = 3].

To further explore the mechanism underlying the redox imbalance and lipid peroxidation in HUVECs exposed to SiNPs, the activities of key anti-oxidative enzymes (including CAT, SOD and GSH-PX) and the MMP [a key biomarker for mitochondrial impairment (28)] were determined. As illustrated in Figure 4A, the red fluorescence produced from J-aggregate in normal mitochondria of HUVECs was clearly weakening with the increasing concentrations of SiNPs exposure, showing a clear reduction in the MMP, which means an increase of mitochondrial impairments. Meanwhile, the activities of the anti-oxidative enzymes (CAT, SOD, and GSH-PX) in HUVECs were decreasing with the elevating concentrations of SiNPs exposure (Figures 4B–D). These results indicated that SiNPs exposure could induce significant increases in both intracellular ROS and lipid peroxidation in HUVECs by both causing mitochondrial impairment and inhibiting the activities of anti-oxidative enzymes (CAT, SOD, and GSH-PX).

### 3.4. p38/Nrf2 pathway

**Figure 5:**
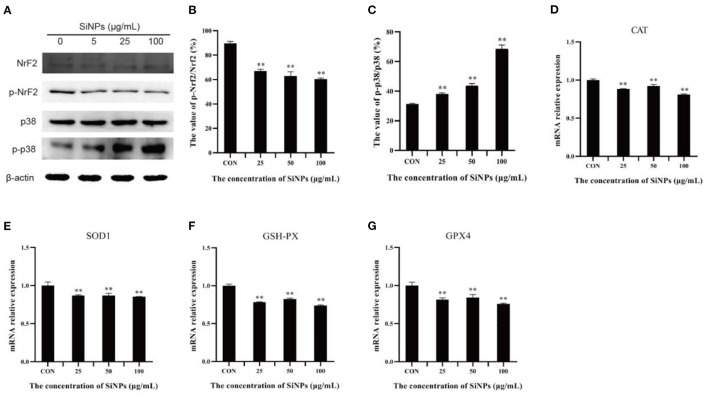
The phosphorylation of p38 and Nrf2 protiens and mRNA expressions of downstream anti-oxidative enzymes. **(A)** the protein bands of NrF2, p-NrF2, p38, and p-p38. **(B)** The relative gray values of p-NrF2 to NrF2. **(C)** The relative gray values of p-p38 to p38. **(D)** The relative mRNA expression of CAT. **(E)** The relative mRNA expression of SOD1. **(F)** The relative mRNA expression of GSH-PX. **(G)** The relative mRNA expression of GPX4. [**p < 0.01, compared with the con, *n* = 3].

To probe the action of transcription factor nuclear factor erythroid-2-related factor 2 (NrF2), a master regulator that moderates the expression of various anti-oxidative enzymes in cells (29), and its upstream inhibitory regulator, the p38 MAPK (mitogen-activated protein kinase) (30), the total NrF2 protein and its phosphorylation (Ser40), total p38 protein and its phosphorylation (Thr180/Tyr182), and the mRNA levels of downstream target genes (anti-oxidative enzymes, such as CAT, SOD1, GSH-PX, and GPX4) were determined with western blot and RT-qPCR respectively. The results showed that the phosphoration of Nrf2 protein (Ser40) was decreasing in HUVECs with the increasing concentrations of SiNPs exposed (Figures 5A, B). Instead, the phosphorylation of p38 (Thr180/Tyr182) was enhancing with the increasing dose of SiNPs exposure (Figures 5A, C). Correspondingly, significant reductions in the mRNA expression of anti-oxidative enzyme genes (CAT, SOD1, GSH-PX, and GPX4) were observed in the SiNPs-treated HUVECs (Figures 5D–G). These data suggested SiNPs exposure may activate the p38 to inhibit the NrF2, resulting in the decreased mRNA expression of downstream antioxidative enzyme genes in HUVECs.

## 4. Discussion

As one of the global top three produced and used NPs, SiNPs have been increasingly exposing to human beings *via* all possible accesses (1, 7). As mentioned above, they will directly expose to the endothelium of cardiovascular system as soon as they pass through the physiological barriers of body or are injected into the bloodstream due to the medical usages (8–10). They will pose adverse effects to cardiovascular system primarily through their toxicities on the ECs, which take a vital role in maintaining the homostasis and normal functions of cardiovascular system as well as in various pathophysiological processes of CVDs (12, 31, 32). But, up to now, our understandings in the adverse effects of SiNPs on ECs, especially about the underlying molecular mechanisms, are still rudimentary.

Ferroptosis is an iron-dependent form of programmed cell death, characterized with mitochondrial impairment, redox imbalance and lipid peroxidation (15). In this study, SiNPs exposure was found to cause a significant decrease in cell viability to HUVECs (Figure 1A), but inhibition of iron ion uptake into cells by DFO (an iron ion chelator) could rescue this reduction in cell viability (Figure 1B), decrease the mRNA expression of both ACSL4 and LPCAT3 [two key enzymes involved in oxidation of polyunsaturated fatty acids in cellular membranes and contribute to ferroptosis (16, 27)], and increase the mRNA expression of GPX4 [a key anti-ferroptotic enzyme (16, 27)] in HUVECs (Figure 2), indicating this SiNPs-mediated cell death in HUVECs is iron-dependent. At the same time, significant increases in mitochondrial impairment (Figure 4A), levels of intracellular ROS and lipid peroxidation (e.g., MDA), as well as a clear reduction in the ratios of GSH/T-GSH (Figure 3), were observed in HUVECs exposed to SiNPs. These results confirmed that SiNPs exposure can induce ferroptosis in HUVECs.

Up to day, the molecular cascades involved in the SiNPs-mediated ferroptosis in HUVECs have not yet been elucidated, while the underlying mechanisms of ferroptosis have been extensively studied since it was first identified (33). Although others (34) and our previous works (1) had shown that SiNPs exposure can induce a significant production of intracellular ROS in the exposed cells, but the underpining mechanisms were not fully clarified. In this study, we found that SiNPs exposure could induce serious redox imbalance with significantly increased intracellular ROS and decreased reductants (e.g., GSH) (Figures 3A–C) in HUVECs *via* multiple approaches, including damaging the mitochondria (Figure 4A), promoting the uptake of iron ion (Figures 1, 2), down-regulating the expression of anti-oxidative enzymes (e.g., CAT, SOD, GSH-PX, and GPX4) (Figures 5D–G) and decreasing their enzymatic activities (Figures 4B–D), resulting in lipid peroxidation (Figure 3D). In addition, SiNPs exposure can also promote lipid peroxidation by both up-regulating the expressions of ACSL4 and LPCAT3 (two critical enzymes for lipid oxidation) and down-regulating the expression of GPX4 (an enzyme can transform lipid hydroperoxides into nontoxic lipid alcohols) (Figure 2). At the same time, the increasing intracellular ROS (e.g., H_2_O_2_) can activate p38 (35) to inhibit NrF2 activation, resulting in reductions in the expressions of the downstream anti-oxidative enzymes (e.g., CAT, SOD, GSH-PX, and GPX4) (Figure 5). Additionally, NrF2 was found to be associated with the outer membrane of mitochondria and protects them from oxidative impairments (36). Thus, this inhibition of NrF2 will further make mitochondria easy to SiNPs-mediated oxidative damaging. Collectively, these have formed a positive feedback for increase in intracellular ROS, mitochondrial impairment and lipid peroxidation, ultimately leading to ferroptosis.

As mentioned above, ECs ferroptosis is an important pathological process involved in AS and its complications (16). This work showed that SiNPs exposure at the tested doses was able to induce ferroptosis in HUVECs. Moreover, Ma et al. (37) have found that amorphous SiNPs can accelerate atherosclerotic lesion progression in ApoE^−/−^ (knockout) mice. These results indicate that long-term and high-dose SiNPs exposure will promote initiation and progression of AS, leading to CVD events. Notably, based on their production and usages, the concentrations of SiNPs in some environmental compartments (e.g., soils and sediments) were estimated to be high to “mg/kg” (38, 39), although there are not yet the real data of human exposure to SiNPs. At “this” level of environmental SiNPs exposure, there will be an increasing atherosclerotic risk for exposed population, especially for those occupational individuals who are more likely to expose to high dose of SiNPs. Induction of ferroptosis in ECs may be an important biological mechanism whereby SiNPs exposure affects the cardiovascular health. EC ferroptosis will be a useful biomarker for assessing the risks of environmental contaminants to the cardiovascular health.

## 5. Conclusion

SiNPs exposure may induce ferroptosis in HUVECs through ROS/p38/NrF2 pathway. Long-term and high-dose SiNPs exposure will increase the atherosclerotic risk to human cardiovascular health. Ferroptosis of ECs will be a useful biomarker for us to assess the potential risks of environmental contaminants to human cardiovascular health.

## Data availability statement

The raw data supporting the conclusions of this article will be made available by the authors, without undue reservation.

## Author contributions

XJ: conceptualization, methodology, investigation, and writing-original draft preparation. HG: conceptualization, methodology, investigation, and writing-reviewing and editing. YC: methodology, data curation, formal analysis, and writing-reviewing and editing. SC, FH, YF, and YZ: investigation and validation. SF: supervision, conceptualization, writing-reviewing and editing, and funding acquisition. All authors contributed to the article and approved the submitted version.
