# Supplementary material for: SiNPs induce ferroptosis in HUVECs through p38 inhibiting NrF2 pathway
Source: Front Public Health. 2023 Feb 8;11:1024130. doi: 10.3389/fpubh.2023.1024130 (PMC9945284; doi:10.3389/fpubh.2023.1024130)
Supplement: Supplementary file 1 [file Data_Sheet_1.pdf]

Table S1 The information of reagents

Reagents	Product #	Company
DMSO	D2650	Sigma, USA
DMEM medium	8121399	Gibco, USA
FBS	2176404	Gibco, South America
HEPES solution	C0215	Beyotime, China
Penicillin-Streptomycin Liquid	P1400	Solarbio, China
CCK-8,Cell Counting Kit-8	CK-04	Dojindo, Japan
DCFH-DA(ROS)	KGT010	KeyGEN, China
MMP(JC-1)	G009-1-3	Jiancheng, China
RIPA	CW2333	Cwbio, China
PVDF	IPVH00010	Millipore, USA
skim milk powder	1172GR100	Biofrox, Germany
eECL Western Blot Kit	CW0049	Cwbio, China
SOD kit	A001-3-2	Jiancheng, China
GSH kit	A006-2-1	Jiancheng, China
Total GSH kit	KGT006	KeyGEN, China
GSH-Px kit	A005-1-2	Jiancheng, China
CAT assay kit	A007-1-1	Jiancheng, China
MDA test kit	A003-1-2	Jiancheng, China
Tissue Total RNA Isolation Kit	RC101	Vazyme, China
cDNA Synthesis Kit	R312-01/02	Vazyme, China
SYBR qPCR Master Mix	Q711	Vazyme, China
Silica nanoparticles	XFI03	XFNANO,China

Table S2 The information of primers

Primers	Forward primer	Reverse primer
actin	CATTCCAAATATGAGATGCGTT	TACACGAAAGCAATGCTATCAC
CAT	TGAAGATGCGGCGAGACTTT	GAGGGGTACTTTCCTGTGGC
SOD1	GCAGATGACTTGGGCAAAGG	TGGGCGATCCCAATTACACC
SOD2	GCACTAGCAGCATGTTGAGC	TTGATGTGAGGTTCCAGGGC
GSH-PX	TATCGAGAATGTGGCGTCCC	TCTTGGCGTTCTCCTGATGC
GPX4	CCGCGATGAGCCTCGG	GGAAAACTCGTGCATGGAGC
ACSL4	GAAGTGAATCGCAGAGTGAA	ACAGAAGATGGCAATGGTG
LPCAT3	TGTGGAAAGACAGGCTGCCA	GGAGTAACCCATGAAGAGCCA
PCBP1	GATTGGGAAAGGCGGGTGTA	CTGCTTGACACACTCGGTGA

Table S3 The information of antibodies

Antibodies	Product #	Company
Anti-ERK1/2	4780	Cell Signal Technology, USA
Anti-phosph-ERK1/2	4370	Cell Signal Technology, USA
Anti-JNK1	3708	Cell Signal Technology, USA
Anti-JNK2	9258	Cell Signal Technology, USA
Anti-phosph-JNK	9255	Cell Signal Technology, USA
Anti-P38 MAPK	8690	Cell Signal Technology, USA
Anti-phosph-P38 MAPK	4511	Cell Signal Technology, USA
Anti-Nrf2	16396-1-AP	Proteintech,USA
Anti-p-Nrf2	ab76026	Abcam,USA
HRP Goat Anti-Rabbit IgG	15015	Proteintech,USA
HRP Goat Anti-mouse IgG	15014	Proteintech,USA
